# Subcutaneous B Cell Lymphoma in a Dog from the West Indies

**DOI:** 10.1155/2020/3695130

**Published:** 2020-01-25

**Authors:** Brittany Cortina, Emily Guenthner, Lara Sypniewski

**Affiliations:** Center for Veterinary Health Sciences, Oklahoma State University's Boren Veterinary Medical Hospital, Stillwater, OK 74074-2041, USA

## Abstract

A 9-year-old male castrated mixed-breed dog from the West Indies was presented for multiple, nonpainful, nodular, circumscribed, subcutaneous masses located on the dorsum, lateral thorax, head, forelimbs, and scrotum. En bloc surgical resection of a mass on the right paw, left forehead, and left medial forelimb with proportional margins was performed. Three punch biopsies were taken from the masses located along the right lateral flank. Histopathologic and immunohistochemistry (IHC) examination of the skin lesions revealed a diagnosis of subcutaneous B cell lymphoma. Thoracic radiographs and abdominal ultrasound were negative for signs of gross metastatic disease. Chemotherapeutic intervention included intravenous doxorubicin (30 mg/m^2^) administered at 3-week intervals for 3 treatments and oral prednisone (2 mg/kg/d) for 3 weeks. There were no complications following the chemotherapy protocol. As of 3 years, there has been no regrowth of the tumors and the patient continues to be cancer free. To date, this is the first reported case of subcutaneous B cell lymphoma diagnosed in a dog treated successfully with gross tumor resection and chemotherapy.

## 1. Introduction

In middle-aged dogs, lymphoma is one of the most common types of cancer diagnosed [1, 2]. Multicentric lymph node involvement is the most common, but other forms include cranial mediastinal, gastrointestinal, cutaneous, and other extranodal forms [2]. Approximately 65-75% of canine multicentric lymphomas are B cell mediated [1]; however, canine lymphoma may also be T cell mediated [2].

Cutaneous and subcutaneous neoplasms involving B or T lymphocytes are rare in canines and felines and account for 1% of canine skin tumors and 2.8% of feline skin tumors [3, 4]. In humans, these tumors have been vastly studied and prognostic factors have been established based on appearance via electron microscopy and immunohistochemical evaluation [5]. Prognostic indicators in canines and felines have been less widely studied, but cutaneous lymphoid neoplasms have been divided into various histopathologic subgroups [6]. With the lack of current information available on the treatment of canine or feline subcutaneous lymphoma, the cutaneous lymphoma subgroups have been used as a guideline for determining the biologic behavior of the disease, and treatment protocols have been extrapolated from alternative and human forms of the disease.

## 2. Case Presentation

A 9-year-old male castrated mixed-breed island dog was presented to Oklahoma State University's Boren Veterinary Medical Hospital (OSU BVMH) for evaluation of several recurrent subcutaneous masses present over the dorsum, lateral thorax, head, forelimbs, and scrotum. Previous medical conditions included chronic thrombocytopenia secondary to Ehrlichia canis (E. Canis) and transmissible venereal tumor (TVT), each acquired while living in the West Indies. Both conditions were responsive to routine treatment. One year prior to presentation to OSU BVMH, the patient was presented to a veterinarian for the presence of multiple subcutaneous masses. Cytologic evaluation of needle aspirates revealed an undifferentiated cutaneous round cell tumor. The masses were removed en bloc, but no histopathologic evaluation was completed. Eight months following this intervention, 10 small dermal masses, roughly one centimeter in diameter, were noted on the dorsum, lateral thorax, head, forelimbs, and scrotum. Four months after the masses were noted, the patient was presented to OSU BVMH for cytologic evaluation which was consistent with a round cell tumor. En bloc mass removal was recommended for further histopathologic evaluation and tumor burden reduction of three of the larger masses. Preoperative blood work revealed mildly elevated liver enzymes but was otherwise fairly unremarkable.

General anesthesia was performed at the discretion of a board certified anesthesiologist, and the subcutaneous masses on the right paw, left forehead, and left medial forelimb were removed en bloc with proportional margins by a senior clinician. The samples were fixed in formalin and submitted to the University of California Davis (UC Davis) Leukocyte Antigen Biology Laboratory for immunohistochemistry (IHC) staining to determine tumor cell origin. Various IHC markers were tested including the leukocyte marker CD18 which was expressed, the T cell marker CD3 which was not expressed, B cell markers CD20 (not expressed) and CD79a (expressed), and histiocytic markers Iba-1, CD204, and E-cadherin, none of which were expressed.

**Figure 1:**
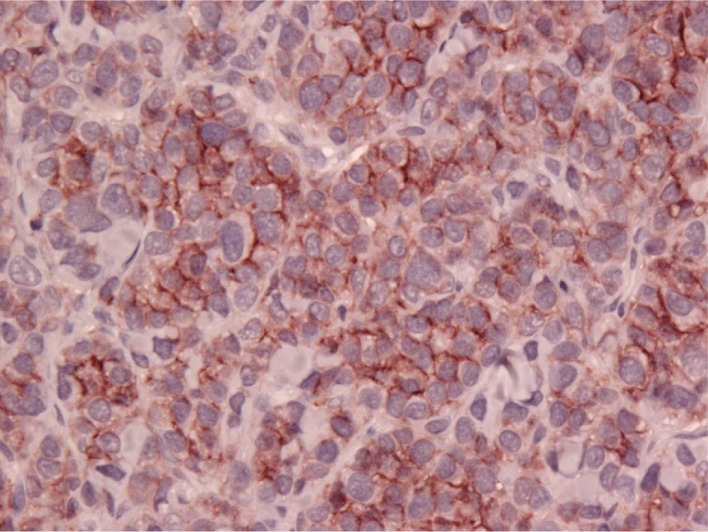
Immunohistochemistry of B cell lymphoma (dog). The tumor cells expressed CD18.

**Figure 2:**
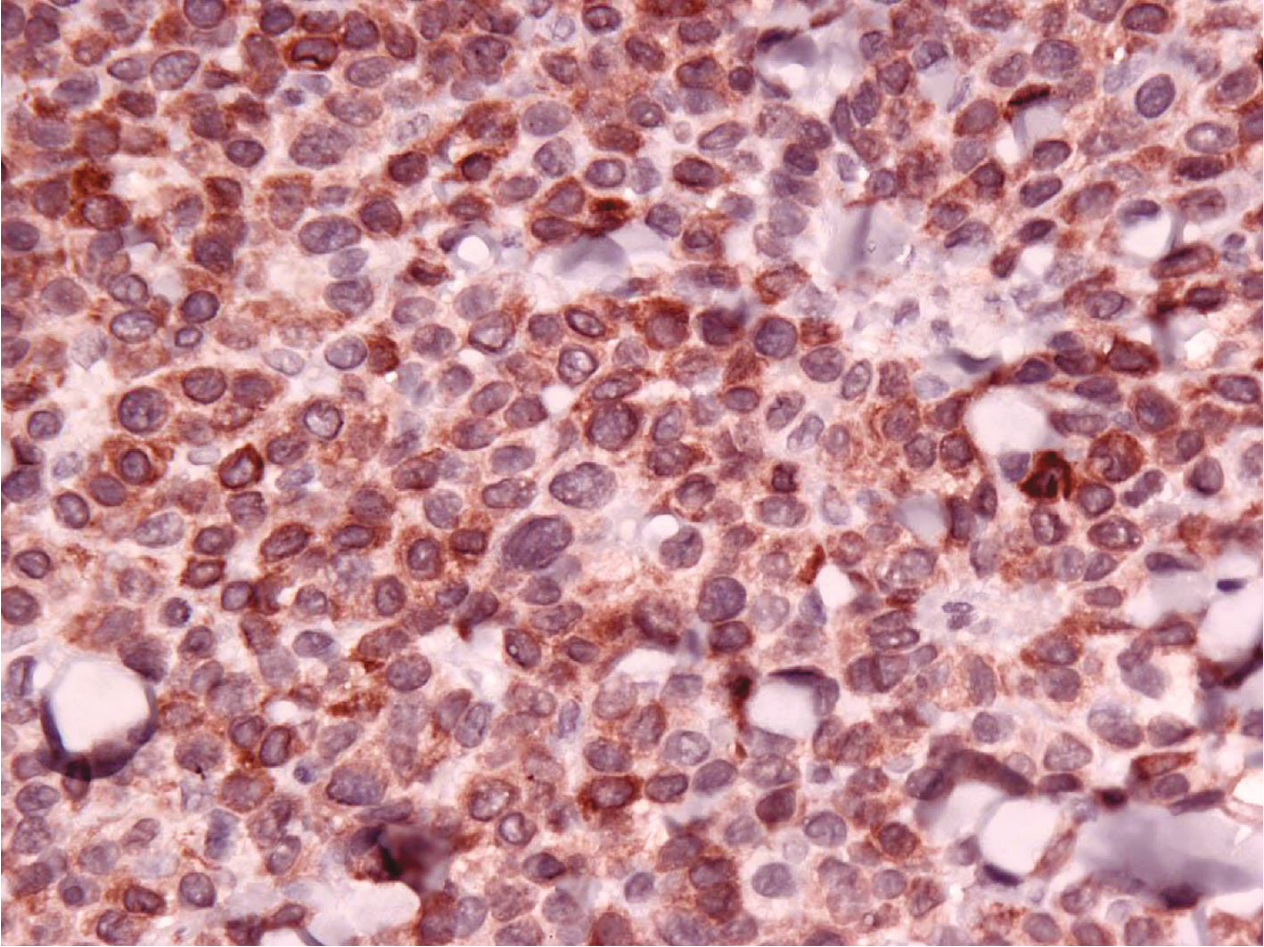
Immunohistochemistry of B cell lymphoma (dog). The subcutis is infiltrated by pleomorphic round cells. The tumor cells expressed the B cell marker CD79a.

A diagnosis of B cell lymphoma was made via IHC and expression of CD79a, which is a B cell antigen receptor complex-associated protein [7]. The specific results of this patient's histopathology report from UC Davis showed large B cell lymphoma with anaplastic cytological features in a subpopulation of tumor cells. Some cells had giant misshapen nuclei, but the majority had large lymphoid morphology, despite the evidence of CD18 expression, which is common for histiocytomas. Atypical cells did not express CD20, a B lymphocyte antigen, but did express CD79a, confirming a B cell origin. Variable nuclear sizes ranging between 3/4 and 10 red blood cells were noted, and these were generally round to ovoid in shape, although some possessed crescentic or misshapen nuclei. There was a high mitotic index 35 per ten 400 × fields. These findings differed from expected IHC findings with canine epitheliotropic lymphoma (CETL) which express CD3, a T cell coreceptor. The results also differed from those expected for a histiocytoma which expresses CD18 and major histocompatibility complex (MHC) II. Although the patient demonstrated the expression of CD18, as seen in Figure 1, the CD79a expression in Figure 2, and lack of MHC II, indicated greater specificity towards B cell lymphoma.

Following IHC diagnosis of subcutaneous B cell lymphoma, the patient was staged with thoracic radiographs and abdominal ultrasound. Thoracic radiographs were reviewed by a board certified radiologist and were clear of evidence of pulmonary metastasis; however, mild sternal lymphadenomegaly was appreciated. Abdominal ultrasound, performed by the same radiologist, showed an enlarged spleen, and splenic fine needle aspirates revealed extramedullary hematopoiesis.

Finances prohibited the recommended treatment for lymphoma with cyclophosphamide, doxorubicin, vincristine, and prednisone protocol. As no gross systemic involvement was noted, single-agent therapy with doxorubicin was instituted. Doxorubicin (30 mg/m^2^) was administered intravenously every 3 weeks for 3 total doses. Oral prednisone (2 mg/kg/day) was prescribed for a one-week induction phase followed by a 3-week tapering dose. Serial complete blood counts (CBC) were evaluated the day of, and 7 days following, doxorubicin administration, with no significant derangements present. The remaining subcutaneous masses that were not removed en bloc regressed during treatment. No new masses were noted following therapy, and follow-up revealed that the patient remains cancer free 3 years following the completion of the medical and surgical treatment.

## 3. Discussion

The etiology of cutaneous lymphoma is unknown [3, 8]. There are several theories as to the predisposing causes for lymphoma, some of which include possible environmental, bacterial, and viral origins [9]. Chronic inflammation caused by bacterial infections has been shown to induce plasma cell proliferation and cancer formation in humans [10]. Few reports in veterinary medicine have demonstrated an association between diseases such as E. Canis, which cause chronic inflammation, and the subsequent development of B cell lymphoma [9] as seen in this particular patient. To the authors' knowledge, there are no reports that describe an association between TVT and the development of lymphoma later in life.

In general, cutaneous lymphomas may be solitary or generalized. They can involve the oral mucosa, lymph nodes, spleen, liver, and bone marrow [8]. There is no apparent breed, sex, or age predilection, but it generally occurs in older dogs [11, 12]. To the authors' knowledge, the biologic behavior of subcutaneous lymphoma of B cell origins has yet to be reported in the dog.

The most commonly described form of cutaneous lymphoma in the veterinary literature is epitheliotropic or of T cell origin [4, 8, 13]. It often targets skin and mucocutaneous junctions and progresses slowly with low-to-intermediate grade malignancy. Dogs may be presented clinically for pruritis and generalized erythema [11, 14, 15]. The other form of the disease is nonepitheliotropic, and it is seen in deeper areas of the dermis and is often of B cell origin [3, 8]. Presenting skin lesions can vary and include generalized scales, plaques, or nodules [3, 8].

Nonepitheliotropic B cell lymphoma, also described as primary cutaneous B cell lymphomas (PCBCL), has been described in humans. Often, PCBCL is specifically localized to the skin, without spread to other organs at the time of diagnosis [16]. PCBCL can be divided into several subcategories including primary cutaneous marginal zone B cell lymphoma (PCMZL), primary cutaneous follicle center lymphoma (PCFCL), and primary cutaneous diffuse large B cell lymphoma (PCDLBCL) leg type (LT), among others [16]. Primary cutaneous marginal zone lymphoma (PCMZL) is one of the more common forms in people and often present as asymptomatic lesions that may be singular or multifocal nodules or papules [16]. In humans, treatment is aimed at surgical excision with concurrent radiation therapy and/or single-agent chemotherapy. Prognosis for both PCFCL and PCMZL overall is very good. Estimated survival rates are approximately 95% or greater at 5 years, with appropriate treatment [16]. On the other hand, PCDLBCL-LT is known to be more aggressive [16].

Conversely, cutaneous epitheliotropic T cell lymphoma (CETL) is known to be a more aggressive disease, often with poor response to treatment and a worse prognosis, depending on the subtype. The three major subtypes described for CETL are mycosis fungoides (MF) which is most common in humans, pagetoid reticulosis (PR), or Sézary syndrome (SS) [3, 14, 17]. In canines, CETL lesions may appear to be highly variable [18]. Lesions initially appear as erythematous patch- or plaque-like structures which consist of scant superficial dermal infiltrates, which then progress to the tumor stage in which the lymphoid infiltrate extends to the reticular dermis and subcutis, leading to progressive ulceration [12, 15]. In humans, non-Hodgkin's lymphoma cases represent approximately 5% of all new cancer cases [12]. Although the CETL in canines closely resembles the human syndrome, in the majority of canine cases, neoplastic cells are CD4-/CD8+ versus CD4+/CD8- in human patients [3, 15]. These cell surface markers are important for differentiating disease subtypes and prognosis [15]. Treatment options have been described to include various chemotherapy-based protocols; however, no improvement in survival time has been noted [15]. The prognosis for survival of CETL is poor in canines [19] with a survival range reported from months to 2 years; this is comparable to humans with CD8+ cytotoxic T cell lymphoma seen with the Sézary form of the disease [15].

To date, there have been no known reports of canine subcutaneous B cell lymphoma in the literature; therefore, diagnosis, staging, and treatment for subcutaneous B cell lymphoma have been determined based on reported treatment successes for CETL in canines and PCBCL in humans. Staging of lymphoma includes collecting a minimum database, consisting of a CBC, chemistry profile, and urinalysis, as well as lymph node aspirate or biopsy with immunophenotyping. Three view thoracic radiographs, abdominal ultrasound, and bone marrow aspirate and cytology are also often recommended as part of the general staging process [2]. Negative prognostic factors include T cell phenotype, mediastinal and gastrointestinal location, “b” or “sick” substage indicating clinical signs of illness, hypercalcemia, poor or incomplete response to treatment, and pretreatment with steroids [2].

Since lymphoma is a systemic disease, treatment for multicentric and cutaneous lymphoma involves systemic chemotherapy [2, 12, 20]. The best response rates have been seen when using multidrug protocols incorporating doxorubicin [1, 2, 12]; however, in cases of cutaneous lymphoma, responses to treatment may be short lived. Surgical excision may also be recommended for solitary nodules [12], and radiation therapy can be used to try to control local disease [6].

The University of Wisconsin-Madison uses a chemotherapy protocol (CHOP) which consists of 3 chemotherapeutic agents (vincristine, cyclophosphamide, and doxorubicin) and a steroid (prednisone). These agents are given on a rotating cycle weekly, for 19 weeks [2]. During this time, prednisone is given orally in a tapering fashion over a 30-day period [2]. For B cell lymphoma, this protocol has the greatest response rates and the longest median survival times [2]. Other protocols report the use of only a single chemotherapeutic agent such as doxorubicin or CCNU (lomustine) and prednisone [2]. A doxorubicin-alone protocol in particular was adapted from the CHOP protocol to include the administration of doxorubicin every 3 weeks for up to 5 treatments in total, for the treatment of some B cell lymphomas [2]. This protocol has somewhat reduced response rates and median survival times, but it may still provide durable remission for some period of time [2]. Prednisone given orally also has some capacity to kill lymphoma cells. Although it is inexpensive and can improve quality of life, oral prednisone's duration of effect is short lived and does not increase median survival time in patients who are on prednisone alone.

Selected treatment protocols vary from patient to patient depending on what is found during initial staging. The doxorubicin protocol with prednisone was the elected treatment course for the patient described, following surgical excision. This particular patient received doxorubicin alone at 30 mg/m^2^, as there was no obvious spread of the disease found upon staging. Doxorubicin treatment was given every 3 weeks for a total of 3 doses. Prednisone was also started at this time and was administered in a tapering fashion. At the end of the chemotherapy protocol, physical exam revealed that the masses had decreased in size and no new masses were noted. The patient described has since had no recurrence of disease and no additional treatment following initial surgery and chemotherapy protocols.

Typical prognosis for CETL is poor, with a mean survival time (MST) of 180-264 days [15]. There is no established MST for B cell subcutaneous lymphoma. We suspect the MST to be similar to that of cutaneous B cell lymphoma in humans and greater than 3 years in canines, based on the outcome of this particular patient.

## 4. Conclusion

Overall, subcutaneous and cutaneous lymphoma is rare in dogs. This report highlights the need to submit all masses and biopsies for histopathology to avoid missing a diagnosis [21]. The signalment, clinical appearance, and presentation of subcutaneous B lymphoma appear to be similar to those of cutaneous T cell lymphoma in canines; however, the biologic behavior of subcutaneous B lymphoma seems to be significantly less aggressive when compared to that of canine cutaneous T cell lymphoma. This disease process most closely resembles that of cutaneous B cell lymphoma in humans. Immunohistochemistry using specific markers is necessary for a definitive diagnosis of this disease. Treatment protocol has been extrapolated from cutaneous lymphoma treatment in humans with surgical resection and single-agent chemotherapy recommended for the best outcome. Further reports of this disease process are warranted to continue to compare and investigate clinical signs, diagnosis, treatment, and prognosis of this disease.

## Abbreviations

IHC: Immunohistochemistry
CETL: Canine epitheliotropic lymphoma
MCH: Major histocompatibility complex
PCBL: Primary cutaneous B cell lymphoma
PCMZL: Primary cutaneous marginal zone B cell lymphoma
LT: Leg type
MF: Mycosis fungoides
PR: Pagetoid reticulosis
SS: Sézary syndrome
CBC: Complete blood count
MST: Mean survival time
TVT: Transmissible venereal tumor
E. Canis: Ehrlichia canis
